# On Mesmerism; or Animal Magnetism

**Published:** 1829-04-01

**Authors:** 


### MESMERISM—ANIMAL MAGNETISM.

A short time ago Mr. Chevenix transmitted to us a paper on the above subject; but, having no department for original communications of that kind, we transmitted it to the Medical and Physical Journal, where it is published. As the author of the paper pledges himself to prove the reality of animal magnetism by ocular demonstration in this country, we deem it right to present our readers with a short outline of the paper in question.

Mr. Chevenix commences somewhat in the Duke of Wellington's style. "ANIMAL MAGNETISM IS TRUE." He next proceeds to adduce the proofs of this assertion. Mr. Chevenix was himself a sceptic—a heretic on this point; but, being now a convert to the true faith, he is perhaps a more zealous propagator of the doctrine than an ordinary believer. While travelling on the Continent, a few years ago, Mr. C. was invited to witness the effects of mesmerism. "I went to laugh —I came away convinced." Mr. C. after being initiated in mesmerism, determined to put it to the test of practice himself, and, in May 1828, he commenced his experiments. The first patient was an epileptic woman, aged 34 years, who had been six years subject to fits, and had, a short time previously, fallen into the fire, and burnt her leg severely. She had daily spasmodic affections and racking pains in different limbs. She was mesmerised several times by Mr. C. and once in the presence of Dr. M'Kay. The consequence, or *apparent* consequence was, that—"in less than a week, the thirst, insomnia, shiverings, and pains, to which she had been subject for six years, ceased —the paralytic tendency diminished—and the spasmodic contractions were entirely removed after the twelfth day of mesmerising." It is proper to state that she was in the 8th month of her sixth pregnancy, when Mr. C. first saw her. The complaint had come on after the first confinement. Mr. C. observes :—"if this case does not offer a fair affiliation of cause and effect, there is no truth in deducing the cure of ague from the administration of bark." We are yet among the sceptics—and candidly tell Mr. Chevenix so; but when he comes over to this metropolis, and fairly throws us into a calm mesmeric slumber "by his will alone, and without any visible manifestation of it," we shall then speak our minds.

We agree with the author that epilepsy "is one of those diseases where the medical art is the most in default." In six months he saw 37 cases, 13 of which were treated by himself, by no other means than mesmerism. "In three of these cases (says he) I was completely successful; in eight more I procured immense relief—two only were failures."

Between the 23d of May, 1828, and the 20th January, 1829, he tried the effects of mesmerism upon 164 persons, of whom 98 manifested undeniable effects—some in one minute—some not until the operation had been several times repeated. "There was hardly an instance where disease existed, that relief was not procured; and many of the patients offered phenomena as extraordinary as any recounted in Germany or France." To shew that mesmerism is a real influence emanating from the mesmeriser, like the galvanic fluid from a trough—and not an imaginary influence or operation in the mesmerised individual, Mr. Chevenix tells us that, "in the presence of the same person, (the gentleman at whose house he was staying,) he mesmerised the patient through the door, and at the distance of fifteen feet—she not knowing that he was acting upon her, but supposing that he was absent." In fourteen minutes she was in complete mesmeric sleep. It would require a good many of such cases to remove our scepticism, knowing how many coincidences are taken for consequences, even where no deception is practised by either party, and no influence of the mind in operation on the patient. We do not deny the reality of Mr. Chevenix's mesmeric influence, merely because we have not had ocular proofs of it. Doubtless—

> There are more things in Heaven and Earth
> Than are dreamt of in our philosophy;—

but we must crave permission to suspend our belief till Mr. C. gives us an opportunity of seeing and examining his experiments. Among at least fifty converts to, and practitioners of mesmerism—for Mr. C. makes no secret of the means, but teaches every one who desires it, the art he himself practises—are, Drs. M'Kay, Cotter, and Peacock—all of them physicians of public establishments in the neighbourhood of the place where the experiments were made. Dr. M'Kay lent his assistance upon all occasions, to determine the nature of the disease, (Mr. C. not being a medical man,) and is ready to testify to the effects of mesmerism; but Dr. Cotter being made of very sceptical materials, the following trial was made.

"On Thursday, October 2d, I requested Dr. Cotter to be present at some mesmeric experiments. He saw two epileptic patients put to sleep in about half a minute each. One of them, while under the mesmeric influence, had a slight fit, which, by increasing the action, I arrested instantaneously. The other he saw me strike motionless, by my will alone, as she walked across the room, and set at liberty in an instant by the same agency. To these facts he, as well as two other gentlemen present, could not refuse their assent; but still a suspicion of connivance and trick might lurk in his mind. I requested him to bring me any five patients of his own, whom he was sure I never could have known or heard of, and to hang his conviction upon this test, that I would, in half an hour, produce effects upon one of these five, which should convince him of the existence of the mesmeric influence.

"On Saturday, October 4th, he came with a female patient, whom he had been treating for dyspepsia, costiveness, and headach, during four years. Her usual aperient dose was thirty grains of jalap with ten of calomel. I never saw her till that day, and only in the presence of Dr. Cotter. She had no idea of what was to be done to her, and was at the moment suffering with severe headache. In three minutes' mesmerising she said her headache was better; in five minutes she said it was quite well. In eight minutes she was in one of the soundest mesmeric sleeps I have witnessed, and continued so for thirty-five minutes, when I awoke her.

"While she was asleep, Dr. Cotter said to me, *in Latin*, that her bowels were at that moment particularly bound. I directed my attention to procuring an evacuation, passing my hands before the abdomen, without, however, touching it, or approaching nearer to it than three or four inches. In less than an hour after she had left the house, she had three evacuations, and for some days her head was considerably relieved. This patient lived at too great a distance for us to continue the treatment; but the following note from Dr. Cotter, relating to another patient, was this moment brought to me:

"'Within this week I have witnessed the effects of mesmerism in the case of Miss P., aged fourteen. She had long been subject to an irregular pain in the left side, over the kidney, accompanied, in its attacks, with a sinking, as described to me, or a tendency to faint. Having long tried medicine without any permanent good, I was desirous to leave off a habit so injurious to a growing subject. I had recourse to mesmerism, without describing to her what I was going to try. Four minutes produced a complete state of somnolency. I have performed it upon this subject but three times, and she has had no return whatever of the pain in her side; neither has there been occasion to exhibit aperients, for which, previously, there was a continual necessity.'"

Mr. Chevenix freely makes known all that he himself knows—he is not a medical man—he practises not for pecuniary or other emolument—he is therefore free from all suspicion or charge of quackery or self-interest. God forbid, then, that we should insinuate any thing like wilful misrepresentation, or knavish design against Mr. Chevenix; but as mesmerism appears to us as miraculous as any of the cures performed by the Author of the Christian Religion, nothing but ocular demonstration can convince us of its reality or truth. The following passage we cheerfully give insertion to.

"I could here enumerate near two hundred examples, but I am fully aware that, in the present condition of the science, these things must be seen to be credited. I shall not, then, attempt to argue or convince my readers, but to implore them to try the experiment themselves. Every one can mesmerise, though not all with equal effect, and practice increases the power: but it is not every one who is susceptible of a sensible influence from this agency; and somnambulism is generally estimated not to occur more frequently than in one case out of five, and not one in twenty-five patients becomes lucid.

"I was myself an unbeliever until I was undeceived by my own experiments: but, had I sooner taken this plain and rational road to knowledge, instead of thinking all men mad who trusted to their eyes that told them truths, which to me seemed more marvellous than all the other wonders of creation, I should, many years since, have possessed the conviction which I now enjoy, and not bewail that, in 1797, my presumptuous ignorance had shut in my own face the door of a science more directly interesting to man than all that chemistry and astronomy can teach. Nine-tenths who may read will laugh at this, as I did at my friend at Rotterdam. Let them do so; but, while they laugh, let them learn, and not, thirty years afterwards, have to lament that so short a remnant of life is left to them to enjoy this new and most valuable secret of Nature."

Whenever Mr. Chevenix comes to London, and mesmerises the patients of public or private practitioners, we shall be happy to bear testimony to the effects of mesmerism. But, till we have ocular demonstration of this strange "secret of Nature," we shall beg leave to continue sceptics, as Mr. Chevenix himself did, before conviction followed irrefragable proof.

